# Supplementary material for: Brain Effects of SC-Nanophytosomes on a Rotenone-Induced Rat Model of Parkinson’s Disease—A Proof of Concept for a Mitochondria-Targeted Therapy
Source: Int J Mol Sci. 2022 Oct 21;23(20):12699. doi: 10.3390/ijms232012699 (PMC9604491; doi:10.3390/ijms232012699)
Supplement: Supplementary file 1 [file ijms-23-12699-s001.zip › ijms-1947566-supplementary.pdf]

Brain effects of *SC*-Nanophytosomes on a rotenone-induced rat model of Parkinson's disease – a proof of concept for a mitochondria-targeted therapy

Daniela Mendes[1], Francisco Peixoto[2], Maria Manuel Oliveira[3], Paula B. Andrade[1] and Romeu A. Videira[1, *]

[1] *REQUIMTE/LAQV*, Laboratory of Pharmacognosy, Department of Chemistry, Faculty of Pharmacy, University of Porto, Rua de Jorge Viterbo Ferreira, nº 228, Porto 4050-313, Portugal.

[2] *Chemistry Center – Vila Real (CQ-VR)*, Biological and Environment Department, School of Life and Environmental Sciences, University of Trás-os-Montes e Alto Douro, UTAD, P.O. Box 1013; 5001-801 Vila Real, Portugal.

[3] *Chemistry Center – Vila Real (CQ-VR)*, Chemistry Department, School of Life and Environmental Sciences, University of Trás-os-Montes e Alto Douro, UTAD, P.O. Box 1013; 5001-801 Vila Real, Portugal.

*Corresponding author: rvideira@ff.up.pt

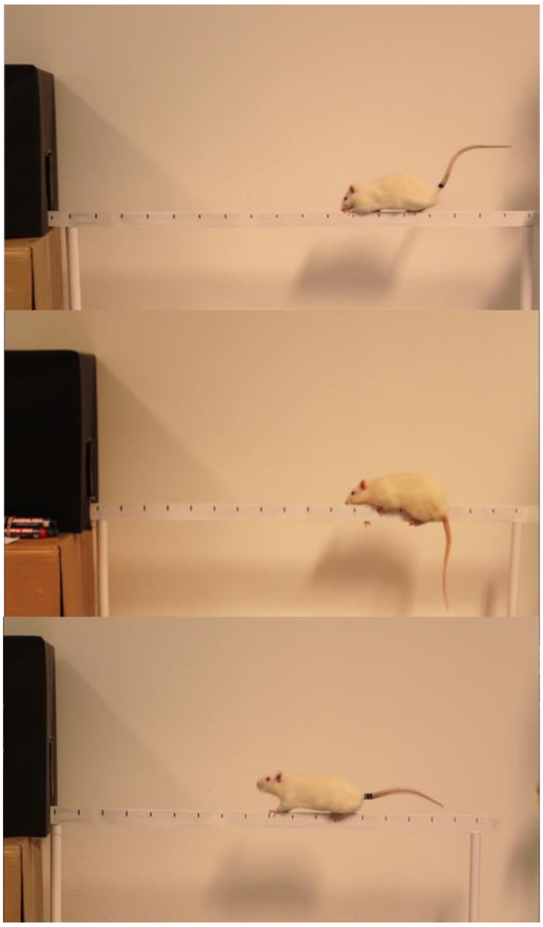

Figure S1. Captions obtained from the video camera records during the beam walking test for the three groups of the experimental plan: CTRL, ROT and ROT+*SC*-Nanophytosomes.

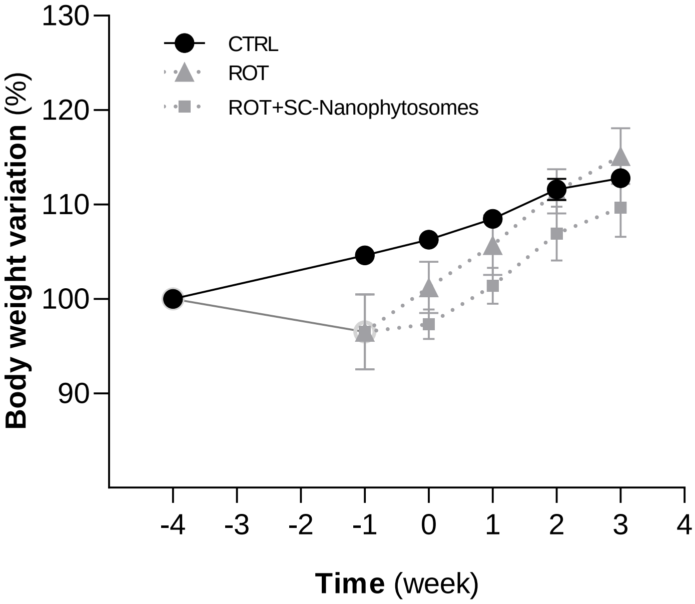

Figure S2. Body weight variation (%) of the rats during the experimental procedure for the CTRL, ROT and ROT+*SC*-Nanophytosomes groups. The animals' body weight, expressed per group by average ±std, when the

study started was: CTRL 397.5±21.4 g and ROT 362.1±32.5 g, and at the end of the study, the average body weight was: CTRL 448.2±22.5 g, ROT 417.5±37.4 g and ROT+*SC*-Nanophytosomes 405.2±19.8 g.

Table S1. Average of the organ weights of Wistar rats from CTRL, ROT and ROT+*SC*-Nanophytosomes groups

**Organ weights** (g)	**CTRL**	**ROT**	**ROT + *SC*-Nanophytosomes**
BG-Cereb	0.868±0.086	0.813±0.030	0.827±0.089
C-Cortex	1.0328±0.136	1.101±0.120	1.018±0.059
Liver	12.638±0.927	12.163±0.907	11.931±1.152
Kidneys	2.648±0.100	2.615±0.283	2.419±0.097
Heart	1.175±0.124	1.210±0.356	1.048±0.080
